# The reverse association between riboflavin intake and Helicobacter pylori infection in US adults: A cross-sectional study

**DOI:** 10.1371/journal.pone.0326787

**Published:** 2025-06-30

**Authors:** Xinyu Xu, Peizhong Chen, Yingyi Li, Xiaoxuan Chen, Yunhui Yan, Zicheng Huang

**Affiliations:** 1 School of Clinical Medicine, Fujian Medical University, Fuzhou, Fujian, China; 2 Department of Gastroenterology, Fujian Medical University Affiliated First Quanzhou Hospital, Quanzhou, Fujian, China; King Abdulaziz University Faculty of Medicine, SAUDI ARABIA

## Abstract

**Objectives:**

This study aimed to examine the association between dietary riboflavin (vitamin B2) intake and Helicobacter pylori infection prevalence among U.S. adults, addressing the growing interest in nutritional strategies for microbial pathogenesis modulation.

**Methods:**

We analyzed data from 2,895 participants in the 1999–2000 National Health and Nutrition Examination Survey (NHANES) with complete dietary and H. pylori serology records. Multivariable logistic regression models adjusted for sociodemographic, clinical, and nutritional covariates were employed to assess riboflavin-infection relationships. Dose-response patterns were evaluated using restricted cubic splines, and subgroup analyses tested heterogeneity across population strata.

**Results:**

The overall H. pylori seropositivity rate was 44.2%. Higher riboflavin intake exhibited a dose-dependent inverse association with infection risk. Compared to the lowest quartile (Q1 ≤ 1.13 mg/day), adjusted odds ratios (95% CI) for Q2 (1.14–1.64 mg/day), Q3 (1.65–2.34 mg/day), and Q4 (≥2.35 mg/day) were 0.77 (0.60–0.99), 0.63 (0.48–0.84), and 0.62 (0.41–0.94), respectively. Spline analysis confirmed a near-linear risk reduction with increasing intake.

**Conclusion:**

These findings suggest that dietary riboflavin may inversely correlate with H. pylori infection in a dose-responsive manner, potentially mediated through mitochondrial function preservation, oxidative stress reduction, and gut microbiota modulation. While observational design precludes causal inference, the results underscore the need for prospective studies evaluating riboflavin’s role in infection prevention.

## 1 Introduction

Helicobacter pylori (H. pylori), a Gram-negative bacterium colonizing the gastric mucosa, is etiologically linked to a spectrum of gastrointestinal disorders, including chronic gastritis and peptic ulcer disease [1]. Global epidemiological data reveal a persistent burden, with 32.8% prevalence reported in the Americas in 2022, highlighting its public health significance [2]. While eradication therapy remains the cornerstone for halting disease progression, emerging antibiotic resistance and recurrence rates necessitate adjunctive preventive approaches. Recent studies have identified dietary factors as potential modulators of H. pylori epidemiology, with observational evidence suggesting associations between specific nutrients and infection prevalence [3–7]. However, the mechanistic role of individual dietary components, including riboflavin, remains poorly characterized, warranting systematic investigation.

Among these candidates, riboflavin (vitamin B2) stands out due to its dual roles in mitochondrial function and antioxidant defense—key pathways targeted by H. pylori virulence factors such as VacA toxin. Experimental evidence indicates that riboflavin deficiency exacerbates gastric oxidative damage and alters gut microbiota composition, both of which may facilitate H. pylori colonization [8–11]. However, population-level evidence linking riboflavin intake to H. pylori infection remains scarce, and the mechanistic contributions of dietary components remain poorly characterized. Our aim was to explore the correlation between riboflavin intake and H. pylori seropositivity, utilizing data from the National Health and Nutrition Examination Survey (NHANES).

## 2 Method

### 2.1 Date sources and study design

The NHANES 1999–2000, conducted by the Centers for Disease Control and Prevention (CDC), provided the observational data for this cross-sectional analysis [12]. Data collection encompassed home interviews, health screenings, and laboratory assessments performed at mobile examination centers (MECs), capturing comprehensive demographic and clinical variables. The original NHANES protocol received approval from the National Center for Health Statistics Ethics Review Panel, with all participants providing written informed consent [13]. Ethical approval for secondary data analysis was waived as the study utilized de-identified publicly available data (accessible at: https://wwwn.cdc.gov/nchs/nhanes/default.aspx).

**Fig 1:**
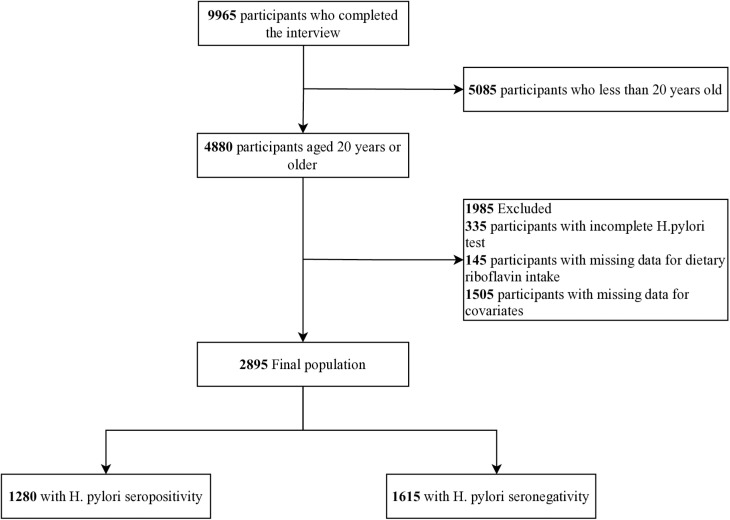
The study’s flow diagram.

From an initial pool of 9,965 eligible participants, exclusion criteria were applied as follows: 5,085 aged <20 years; 335 with incomplete H. pylori serology; 145 lacking dietary riboflavin data; and 1,505 with insufficient covariate information. Participants with missing data were excluded to ensure analytical integrity. After exclusions, 2,895 participants comprised the final analytical cohort. A detailed flowchart of inclusion and exclusion criteria is presented in Fig 1 with supplementary shows comparison of basic characteristics between excluded and included populations (S1 Table). This study adhered to the Strengthening the Reporting of Observational Studies in Epidemiology (STROBE) guidelines for cross-sectional research.

### 2.2 Dietary riboflavin intake

Dietary intake in NHANES was evaluated through two 24-hour recalls [14]. The first recall was conducted in-person at the MEC by trained interviewers, followed by a remote session (telephone or mail) within 3–10 days. Total riboflavin intake, encompassing both dietary and supplemental sources, was calculated as the average of both recalls per NHANES protocols [15]. Data collection utilized the NHANES Computer-Assisted Dietary Interview System (CADI), a multi-pass method that standardizes interviewer instructions for granular dietary reporting. All nutrient values were coded using the USDA’s Food Composition Database to ensure consistency.

Riboflavin intake was evaluated both as continuous measurements and categorical groupings (Q1–Q4). The participants were divided into four parts according to their dietary riboflavin intake: Q1 (≤1.13 mg/day), Q2 (1.14–1.64 mg/day), Q3 (1.65–2.34 mg/day), and Q4 (≥2.35 mg/day).

### 2.3 Helicobacter pylori antibody measurement

H. pylori-specific IgG antibodies in serum were quantified via ELISA (Wampole Laboratories) [16]. According to manufacturer guidelines, seropositivity was defined as optical density (OD) ≥0.9, with OD < 0.9 classified as seronegative [17].

### 2.4 Covariates

Covariates were selected based on prior literature [6,16–21] and categorized into four domains:

demographics: age (continuous), sex (male/female), education (<12, = 12, > 12 years), marital status, family income (low/medium/high based on U.S. government report [22]);lifestyle factors: smoking status (never-smoker: < 100 cigarettes during lifetime; smoker: ≥ 100 cigarettes [23]), alcohol consumption (never drinker: life alcohol intake<12 drinks; drinker: ≥ 12 drinks [23]), BMI (<25 kg/m^2^ [normal weight], 25–30 kg/m^2^ [overweight], ≥ 30 kg/m^2^ [obese] [24]);clinical characteristics: self-reported physician-diagnosed diabetes and cardiovascular conditions (hypertension, angina, coronary artery disease, myocardial infarction, heart failure, stroke), serum biomarkers (creatinine, C-reactive protein [CRP], albumin, total cholesterol). dietary factors: total caloric intake, macronutrients (carbohydrates, dietary fiber), micronutrients (vitamins B1, B6, B12, C, A, E, carotene, niacin, folate; minerals: calcium, phosphorus, iron, zinc, sodium, potassium), and dietary supplement use (past 30 days), collected via 24-hour dietary recall interviews.

### 2.5 Statistical analysis

This secondary analysis utilized data from the NHANES 1999–2000. Continuous variables were summarized as mean ± standard deviation (SD) for normally distributed data or median (interquartile range, IQR) for non-normally distributed data, while categorical variables were expressed as frequencies (%). Group comparisons were performed using: One-way analysis of variance (ANOVA) for normally distributed continuous variables, Kruskal-Wallis test for non-normally distributed continuous variables and Chi-square test for categorical variables.

To evaluate the association between dietary riboflavin intake and H. pylori seropositivity, multivariable logistic regression models were constructed with progressive covariate adjustment: Model 1: Adjusted for sociodemographic variables (age, sex, education level, marital status, family income);Model 2: Model 1 + lifestyle and clinical variables (BMI, smoking status, alcohol consumption, diabetes, cardiovascular diseases) and serum biomarkers (creatinine, CRP, albumin, total cholesterol);Model 3: Model 2 + dietary covariates (total caloric intake, carbohydrates, dietary fiber, vitamins B1/B6/B12/C/A/E, carotene, niacin, folate, calcium, phosphorus, iron, zinc, sodium, potassium, and dietary supplements). Dietary riboflavin intake was analyzed both as a continuous variable (per 1 mg/day increment) and as quartiles, with Q1 as the reference. A linear trend test across quartiles was performed by assigning median values to each quartile and modeling them as a continuous variable.

Subgroup analyses evaluated potential modifiers (sex, age, marital status, education, income, BMI, supplement use) in the riboflavin–H. pylori association. Interaction terms were tested via hierarchical logistic regression, with significance determined by likelihood ratio tests and Wald statistics.

For sensitivity testing, participants with extreme calorie intake (top and bottom 1%, *n* = 58) were excluded to verify result stability. All subgroup models retained adjustments for covariates specified in the primary analysis. Moreover, we used multiple imputation, based on 3 replications and a chained equation approach method in the R mice procedure, to maximize statistical power and minimize bias that might occur account for missing data. We also performed sensitivity analyses using a multiple imputation analysis. All statistical analyses were performed using R software (version 4.2.2; R Foundation for Statistical Computing). Statistical significance was defined as a two-sided P < 0.05, with results reported as odds ratios (ORs) and 95% confidence intervals (CIs).

## 3 Results

### 3.1 Baseline characteristics

Demographic and baseline attributes of the enrolled and excluded subjects are provided in S1 Table. Baseline characteristics of the study population, stratified by riboflavin intake quartiles, are summarized in Table 1 of the 2,895 participants, 1,280 (44.2%) were seropositive for H. pylori. Participants in higher riboflavin intake quartiles tended to be younger, male, and married or cohabiting. They also demonstrated higher educational attainment, greater family income, and lower BMI. Furthermore, this group exhibited a lower prevalence of diabetes and cardiovascular diseases, including hypertension, angina, coronary artery disease, heart attack, heart failure and stroke. Finally, individuals in higher intake quartiles reported greater caloric intake, higher use of dietary supplements, and a higher proportion of alcohol consumers compared to lower quartiles.

**Table 1:** Population characteristics by categories of dietary riboflavin intake.

Characteristic	Riboflavin intake, mg/d					
	Total	Q1(≤1.13)	Q2(1.14–1.64)	Q3(1.65–2.34)	Q4(≥2.35)	p
NO.	2895	715	729	726	725	
Age (year), Mean (SD)	49.9 ± 18.7	51.7 ± 18.6	51.0 ± 18.5	49.9 ± 18.6	46.8 ± 18.6	<0.001
Sex, n (%)						<0.001
Male	1374 (47.5)	236 (33)	307 (42.1)	376 (51.8)	455 (62.8)	
Female	1521 (52.5)	479 (67)	422 (57.9)	350 (48.2)	270 (37.2)	
Education level (year), n (%)						<0.001
<12	1043 (36.0)	339 (47.4)	273 (37.4)	235 (32.4)	196 (27)	
=12	659 (22.8)	153 (21.4)	169 (23.2)	169 (23.3)	168 (23.2)	
>12	1193 (41.2)	223 (31.2)	287 (39.4)	322 (44.4)	361 (49.8)	
Marital status, n (%)						<0.001
Living alone	1068 (36.9)	329 (46)	268 (36.8)	242 (33.3)	229 (31.6)	
Married or living with a partner	1827 (63.1)	386 (54)	461 (63.2)	484 (66.7)	496 (68.4)	
Family income, n (%)						<0.001
Low	868 (30.0)	283 (39.6)	232 (31.8)	186 (25.6)	167 (23)	
Medium	1093 (37.8)	272 (38)	271 (37.2)	272 (37.5)	278 (38.3)	
High	934 (32.3)	160 (22.4)	226 (31)	268 (36.9)	280 (38.6)	
Body mass index (kg/m2), n (%)						0.013
<25	897 (31.0)	203 (28.4)	213 (29.2)	220 (30.3)	261 (36)	
≥25, < 30	1041 (36.0)	255 (35.7)	274 (37.6)	254 (35)	258 (35.6)	
≥30	957 (33.1)	257 (35.9)	242 (33.2)	252 (34.7)	206 (28.4)	
Smoker, n (%)	1384 (47.8)	306 (42.8)	340 (46.6)	389 (53.6)	349 (48.1)	<0.001
Drinker, n (%)	1948 (67.3)	405 (56.6)	493 (67.6)	522 (71.9)	528 (72.8)	<0.001
Diabetes, n (%)	269 (9.3)	75 (10.5)	78 (10.7)	68 (9.4)	48 (6.6)	0.028
Hypertension, n (%)	891 (30.8)	237 (33.1)	257 (35.3)	224 (30.9)	173 (23.9)	<0.001
Heart failure, n (%)	85 (2.9)	35 (4.9)	20 (2.7)	18 (2.5)	12 (1.7)	0.002
Coronary disease, n (%)	114 (3.9)	29 (4.1)	31 (4.3)	30 (4.1)	24 (3.3)	0.791
Angina, n (%)	119 (4.1)	30 (4.2)	34 (4.7)	29 (4)	26 (3.6)	0.775
Heart attack, n (%)	125 (4.3)	36 (5)	38 (5.2)	26 (3.6)	25 (3.4)	0.205
Stroke, n (%)	89 (3.1)	33 (4.6)	20 (2.7)	22 (3)	14 (1.9)	0.027
Serum indicators						
Creatinine (mg/dL), Median (IQR)	0.7 (0.6, 0.9)	0.7 (0.5, 0.8)	0.7 (0.6, 0.8)	0.7 (0.6, 0.9)	0.7 (0.6, 0.9)	0.008
C reactive protein (mg/dl), Median (IQR)	0.3 (0.1, 0.6)	0.3 (0.1, 0.7)	0.3 (0.1, 0.6)	0.2 (0.1, 0.5)	0.2 (0.1, 0.5)	<0.001
Albumin (g/dL), Mean ±SD	4.4 ± 0.3	4.4 ± 0.3	4.4 ± 0.3	4.4 ± 0.3	4.4 ± 0.4	0.005
Total cholesterol (mg/dL), Mean ± SD	198.9 ± 39.7	200.7 ± 41.9	198.8 ± 37.8	197.6 ± 38.1	198.7 ± 40.9	0.523
Calorie consumption (kcal/d), Mean ± SD	2072.3 ±1010.0	1240.0 ± 518.4	1782.4 ± 568.6	2215.8 ± 699.5	3040.9 ±1126.3	<0.001
Carbohydrate consumption (gm/d),Median (IQR)	237.7(166.8, 325.4)	158.3(109.8, 204.5)	211.8(157.7, 269.4)	262.6(202.9, 328.2)	353.0(284.0, 451.1)	<0.001
Dietary fiber consumption (gm/d), Median (IQR)	13.4 (8.7, 20.2)	8.2 (5.2, 12.2)	12.6 (8.5, 17.8)	15.1 (10.4,21.1)	20.4 (14.1,28.4)	<0.001
Dietary supplements taken, n (%)	1469(50.7)	300 (42)	363 (49.8)	376 (51.8)	430 (59.3)	<0.001
VitaminB1 intake (mg/d), Median (IQR)	1.4 (0.9, 2.0)	0.8 (0.5, 1.0)	1.2 (0.9, 1.5)	1.6 (1.3, 1.9)	2.4 (1.9, 3.0)	<0.001
VitaminB6 intake (mg/d), Median (IQR)	1.6 (1.0, 2.3)	0.9 (0.6, 1.3)	1.4 (1.0, 1.8)	1.8 (1.4, 2.3)	2.7 (2.1, 3.7)	<0.001
Vitamin C intake (mg/d), Median (IQR)	71.1 (31.7, 139.9)	39.9 (14.9, 90.9)	64.3 (29.5, 122.6)	77.1 (38.1, 147.6)	110.9 (58.5, 200.3)	<0.001
Vitamin A intake (RE/d), Median (IQR)	625.7(333.1, 1153.6)	262.9(144.9, 501.3)	468.1 (318.8, 787.7)	713.5(496.8, 1156.8)	1197.7(803.1, 1837.9)	<0.001
Carotene intake (RE/d), Median (IQR)	191.7 (78.4, 514.4)	113.2 (40.7, 323.8)	185.5 (73.9, 468.9)	230.6 (96.5, 626.1)	255.3 (127.8, 670.4)	<0.001
Vitamin E intake (mg/d), Median (IQR)	7.0 (4.5, 10.6)	4.2 (2.7, 6.0)	6.4 (4.4, 9.1)	8.1 (5.8, 10.8)	11.0(7.3, 16.1)	<0.001
Niacin intake (mg/d), Median (IQR)	19.5 (13.5,27.7)	11.8 (8.3, 15.6)	16.6 (13.2,22.5)	21.4 (17.1,27.3)	31.2 (23.7,41.0)	<0.001
Folate intake (mcg/d), Median (IQR)	320.8 (213.9, 467.0)	176.8 (124.6, 245.9)	276.6 (213.1, 352.3)	362.8 (285.5, 458.1)	539.7 (413.3, 704.2)	<0.001
VitaminB12 intake (mcg/d), Median (IQR)	3.3 (1.8, 5.3)	1.4 (0.7, 2.6)	2.7 (1.7, 4.0)	3.7 (2.4, 5.3)	6.0 (4.1, 9.0)	<0.001
Calcium intake (mg/d), Median (IQR)	654.9 (403.9, 1037.1)	296.5(200.7, 425.2)	551.9(416.6, 718.3)	792.3(611.9, 1030.2)	1292.8 (973.7,1648.0)	<0.001
Phosphorus intake (mg/d), Median (IQR)	1127.3(798.7, 1574.9)	616.7(465.0, 773.7)	985.0(836.5, 1149.3)	1283.4(1097.5, 1531.6)	1876.8(1574.9, 2255.2)	<0.001
Iron intake (mg/d), Median (IQR)	12.7 (8.8, 18.4)	7.4 (5.4, 9.4)	11.1 (8.9, 13.9)	14.5 (11.6,18.6)	22.2 (16.9,30.0)	<0.001
Zinc intake (mg/d), Median (IQR)	9.3(6.3, 13.5)	5.1(3.5, 7.1)	8.1(6.4, 10.7)	10.4 (8.2,13.6)	15.4 (11.9, 21.2)	<0.001
Sodium intake (mg/d), Median (IQR)	2916.1(1987.2, 4141.7)	1751.6 (1199.2, 2402.4)	2625.8 (1960.4, 3474.9)	3346.0(2506.4, 4233.3)	4428.8(3261.0, 5914.1)	<0.001
Potassium intake (mg/d), Median (IQR)	2487.2(1724.9, 3409.2)	1451.0(1027.6, 1983.1)	2231.8(1724.3, 2779.0)	2824.9(2279.1, 3411.7)	3823.4(3035.6, 4709.0)	<0.001
H. pylori seropositivity, n (%)	1280 (44.2)	393 (55)	337 (46.2)	291 (40.1)	259 (35.7)	<0.001

### 3.2 Association between dietary riboflavin intake and H. pylori seropositivity

Univariate analysis identified multiple variables associated with H. pylori seropositivity (S2 Table), including demographic factors (age, education level, family income), lifestyle markers (BMI, smoking, alcohol use), clinical conditions (diabetes, hypertension, heart failure, myocardial infarction, stroke), serum albumin, and dietary components (caloric intake, carbohydrates, vitamins B1/B6/A/E, niacin, folate, B12, calcium, phosphorus, iron, zinc, sodium, potassium, and supplement use).

**Fig 2:**
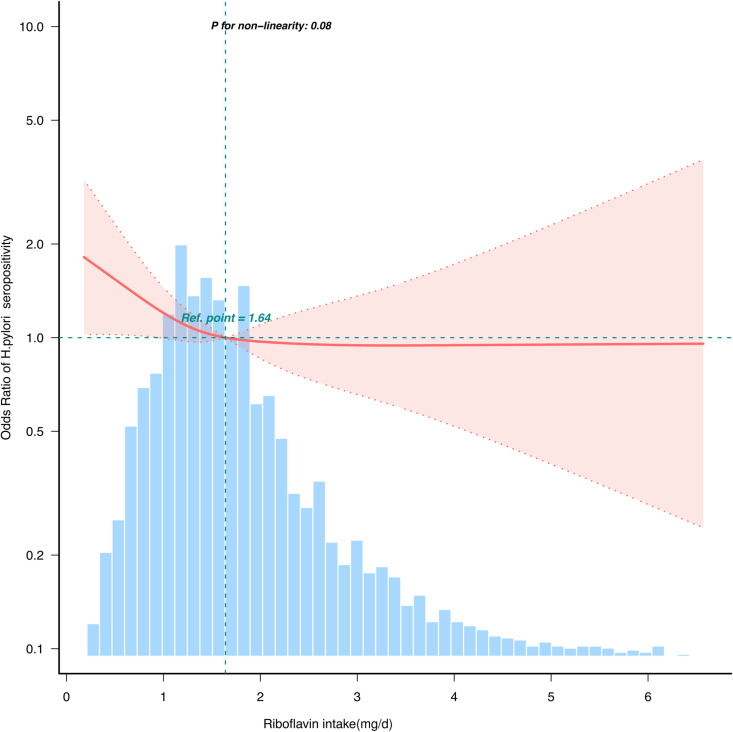
Association between dietary riboflavin intake and H. pylori seropositivity odds ratio. Solid and dashed lines represent the predicted value and 95% CIs. They were adjusted for sociodemographic variables (age, sex, education level, marital status, family income) lifestyle and clinical variables (BMI, smoking status, alcohol consumption, diabetes, cardiovascular diseases) and serum biomarkers (creatinine, CRP, albumin, total cholesterol) dietary covariates (total caloric intake, carbohydrates, dietary fiber, vitamins B1/B6/B12/C/A/E, carotene, niacin, folate, calcium, phosphorus, iron, zinc, sodium, potassium, and dietary supplements). Only 99% of the data is shown.

In multivariable models, dietary riboflavin intake exhibited a significant inverse dose-response relationship with H. pylori seropositivity (**Table 2**). Compared to the lowest quartile (Q1 ≤ 1.13 mg/day), adjusted ORs for higher quartiles were:Q2 (1.14–1.64 mg/day) 0.77(95% CI: 0.60–0.99), Q3 (1.65–2.34 mg/day) 0.63 (95% CI: 0.48–0.84), Q4 (≥2.35 mg/day)0.62(95% CI: 0.41–0.94). This trend was further supported by smoothed curve analysis, which revealed a near-linear negative association between riboflavin intake and infection probability (**Fig 2**).

**Table 2:** Multivariable logistic regression to assess the association of riboflavin intake with Helicobacter pylori seropositivity.

Variable	OR (95%CI)							
	Crude	p-value	Model1	p-value	Model 2	p-value	Model3	p-value
Riboflavin intake (mg/day)								
	0.78 (0.73 ~ 0.84)	<0.001	0.86 (0.8 ~ 0.93)	<0.001	0.85 (0.79 ~ 0.92)	<0.001	0.9 (0.73 ~ 1.13)	0.376
Q1(≤1.13)	1(Ref)		1(Ref)		1(Ref)		1(Ref)	
Q2(1.14–1.64)	0.7 (0.57 ~ 0.87)	0.001	0.79 (0.63 ~ 0.99)	0.037	0.78 (0.62 ~ 0.97)	0.028	0.77 (0.6 ~ 0.99)	0.037
Q3(1.65–2.34)	0.55 (0.44 ~ 0.68)	<0.001	0.65 (0.52 ~ 0.82)	<0.001	0.64 (0.51 ~ 0.81)	<0.001	0.63 (0.48 ~ 0.84)	0.002
Q4(≥2.35)	0.46 (0.37 ~ 0.56)	<0.001	0.59 (0.47 ~ 0.75)	<0.001	0.58 (0.46 ~ 0.74)	<0.001	0.62 (0.41 ~ 0.94)	0.024
Trend test		<0.001		<0.001		<0.001		0.0046

Q, quartiles; OR, odds ratio; CI, confidence interval; Ref: reference.

Model 1: Adjusted for sociodemographic variables (age, sex, education level, marital status, family income);

Model 2: Model 1 + lifestyle and clinical variables (BMI, smoking status, alcohol consumption, diabetes, cardiovascular diseases) and serum biomarkers (creatinine, CRP, albumin, total cholesterol)

Model 3: Model 2 + dietary covariates (total caloric intake, carbohydrates, dietary fiber, vitamins B1/B6/B12/C/A/E, carotene, niacin, folate, calcium, phosphorus, iron, zinc, sodium, potassium, and dietary supplements).

### 3.3 Subgroup analyses

**Fig 3:**
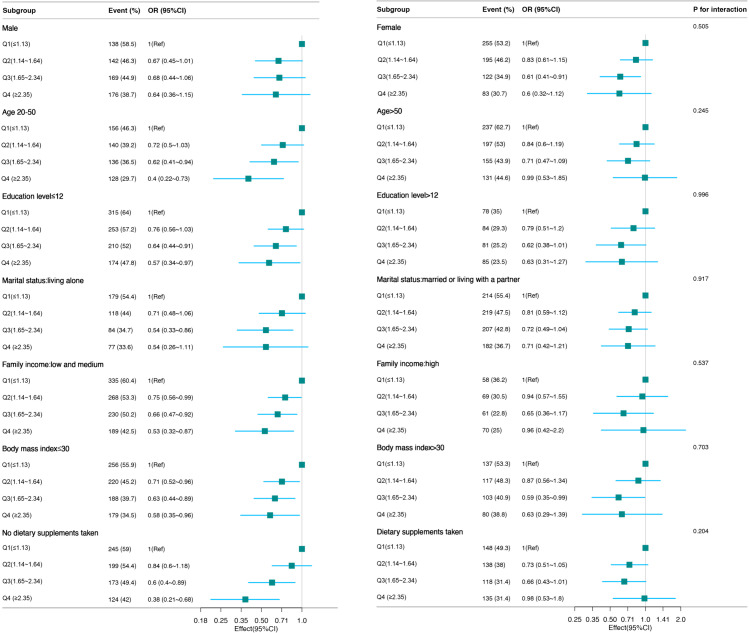
The relationship between dietary riboflavin intake and Helicobacter pylori seropositivity. Except for the stratification component itself, each stratification factor was adjusted for all other variables: sociodemographic variables (age, sex, education level, marital status, family income) lifestyle and clinical variables (BMI, smoking status, alcohol consumption, diabetes, cardiovascular diseases) and serum biomarkers (creatinine, CRP, albumin, total cholesterol) dietary covariates (total caloric intake, carbohydrates, dietary fiber, vitamins B1/B6/B12/C/A/E, carotene, niacin, folate, calcium, phosphorus, iron, zinc, sodium, potassium, and dietary supplements).

Subgroup analyses were performed according to stratification variables: sex, age, family income, marital status, education level, BMI, dietary supplements taken. Forest plots visualizing these subgroup analyses revealed no statistically significant interactions (**Fig 3**).

### 3.4 Sensitivity analyses

Sensitivity analyses demonstrated consistent inverse associations between higher riboflavin intake and H. pylori seropositivity. After excluding extreme caloric intake (N = 2,837 remaining), adjusted ORs for quartiles Q3 (1.65–2.34 mg/day) and Q4 (≥2.35 mg/day) were 0.72 (95% CI: 0.53–0.97) and 0.74 (95% CI: 0.47–1.15), respectively, compared to Q1 (≤1.13 mg/day) (S3 Table). Multiple imputation incorporating missing data (N = 4,000) yielded similar trends, with ORs of 0.73 (0.57–0.92) for Q3 and 0.78 (0.55–1.11) for Q4 (S4–S6 Tables). While Q4 estimates showed wider confidence intervals overlapping the null, the persistent risk reduction in Q3 across both analyses supports the robustness of the inverse association.

## 4 Discussion

This cross-sectional study identified a robust inverse association between dietary riboflavin intake and H. pylori seropositivity, with robustness confirmed through stratified and sensitivity analyses. While antibiotic therapy remains the primary treatment, our findings suggest dietary riboflavin levels may influence infection epidemiology. Given that riboflavin cannot be synthesized endogenously [25] and excess amounts are rapidly metabolized and renally excreted, consistent dietary intake is critical. This metabolic dependency may explain population-level variability in infection prevalence. Public health initiatives should prioritize community education on riboflavin’s chemopreventive mechanisms and integrate its fortification into H. pylori control programs. Further research must validate dose-dependent preventive effects and therapeutic synergies with antimicrobial regimens to optimize evidence-based guidelines.

To our knowledge, this is the first effort to examine the correlation between H. pylori seropositivity and dietary riboflavin intake. Although Maki Inoue-Choi et al. [26] proposed vitamin B as a chemopreventive agent for H. pylori-related gastropathy, our findings extend this paradigm by directly linking riboflavin to infection susceptibility. Given that >75% of gastric cancer cases are attributed to H. pylori [27,28], riboflavin’s dual role as an antioxidant and anti-carcinogen [29,30] gains clinical relevance. Specifically, by attenuating oxidative stress and mitochondrial dysfunction—key drivers of H. pylori virulence—riboflavin may disrupt infection pathways. Therefore, optimizing riboflavin intake could serve as a dual-purpose strategy: influencing primary infection and mitigating carcinogenic sequelae.

This study examines the relationship between riboflavin intake and H. pylori susceptibility, with particular attention to mechanisticaspects that have received relatively little attention in prior research. While the exact mechanisms require further investigation, the integration of epidemiological and molecular insights strengthens the hypothesis that riboflavin deficiency exacerbates infection risk.

Experimental studies demonstrate that VacA, a H. pylori virulence factor critical for colonization [31,32], induces mitochondrial dysfunction through outer membrane permeabilization (MOMP) and dissipation of the mitochondrial membrane potential (ΔΨm) [33,34]. Mitochondrial integrity is essential for gastric epithelial homeostasis, as these organelles regulate ATP-dependent cellular processes. VacA-mediated energy depletion has been linked to enhanced bacterial colonization in vitro [35]. Interestingly, higher riboflavin intake in our study coincided with lower H. pylori seropositivity. Riboflavin serves as a precursor for FAD and FMN—cofactors integral to electron transport chain (ETC) function. Since ΔΨm maintenance depends on ETC efficiency [9–11], riboflavin-associated FAD availability could theoretically mitigate VacA-induced mitochondrial perturbations.

Oxidative stress is mechanistically linked to H. pylori pathogenesis, as evidenced by both clinical and experimental studies. Riboflavin plays a dual role in cellular antioxidant systems, primarily through the glutathione redox cycle. In this cycle, glutathione peroxidase (GPx) catalyzes the conversion of reduced glutathione (GSH) to oxidized glutathione disulfide (GSSG), neutralizing hydrogen peroxide (H₂O₂) and lipid peroxides. Glutathione reductase (GR) then utilizes riboflavin-derived FAD to regenerate GSH from GSSG, sustaining redox balance. Additionally, riboflavin may modulate antioxidant enzyme expression. In vitro studies report upregulated SOD, GPx, and CAT levels in riboflavin-treated stromal cells [36], implying a role in redox homeostasis, though direct evidence in gastric epithelium remains limited. Riboflavin also exhibits radical-scavenging capacity via redox cycling between dihydro- and oxidized forms [37]. Experimental models further suggest riboflavin synergizes with vitamins C and E to amplify antioxidant defenses, as observed in rodent neural tissue [38].

Emerging evidence suggests riboflavin intake may correlate with gut microbiota composition, which could hypothetically influence H. pylori colonization. Preclinical studies demonstrate that riboflavin supplementation alters murine gut microbiota β-diversity [39], while a clinical trial in healthy adults reported increased *Faecalibacterium prausnitzii* abundance after 2-week riboflavin intervention [40]. Additionally, observational data reveal an inverse association between dietary riboflavin and *Streptococcus spp.* colonization in humans [41], suggesting riboflavin-associated microbiota profiles may coincide with reduced pathogen persistence. Although above mechanisms provide a plausible framework for our observed inverse riboflavin-H. pylori association, future work should employ in vitro models (e.g., VacA-treated gastric epithelial cells) or interventional animal studies to elucidate riboflavin’s effects on mitochondrial function, oxidative stress, and gut microbiota composition.

While this study provides novel insights into the relationship between dietary riboflavin and H. pylori seropositivity, several limitations warrant acknowledgment. First, as IgG antibodies cannot differentiate between active infection and prior exposure, future investigations should adopt direct diagnostic approaches—such as urea breath tests, stool antigen assays, or endoscopic biopsies—to confirm active bacterial colonization and improve the precision of dietary risk assessments. Second, dietary intake was evaluated using the Food Frequency Questionnaire (FFQ), which may introduce recall bias and underreporting, particularly among symptomatic individuals, who may have modified their dietary habits due to H. pylori-related gastrointestinal discomfort. Furthermore, the FFQ does not account for intra-individual seasonal variations in diet or the influence of food preparation methods on riboflavin bioavailability. To mitigate these issues, subsequent studies should incorporate biomarker validation (e.g., urinary riboflavin quantification) and granular data on culinary practices to enhance dietary assessment accuracy. Third, the cross-sectional design precludes causal inference, leaving the directionality of the association unresolved. It remains unclear whether riboflavin intake modulates H. pylori seropositivity or whether infection-driven avoidance of riboflavin-rich foods (e.g., due to symptom exacerbation) confounds the relationship. Although sensitivity analyses adjusted for major confounders, residual confounding from unmeasured variables—such as genetic predispositions, medication use, or unrecorded dietary components—cannot be fully excluded. Prospective cohort studies, randomized controlled trials, or Mendelian randomization analyses utilizing genetic proxies for riboflavin metabolism are essential to elucidate causality. Fourth, Although the data are historical, the inverse association between riboflavin intake and H. pylori seropositivity is grounded in biological mechanisms that are temporally invariant. Replication in modern cohorts with updated serological and dietary datasets is imperative to confirm the robustness of these associations. Finally, the generalizability of our conclusions is constrained by the restriction of the study sample to U.S. adults and the absence of weighted adjustments for population diversity. Multicenter studies spanning diverse geographic regions and demographic groups are necessary to validate the translational applicability of these findings.

## 5 Conclusion

In conclusion, this study highlights an inverse association between dietary riboflavin intake and H. pylori prevalence underpinned by plausible mechanisms involving mitochondrial function, cellular oxidative stress and gut microbiota composition. Nonetheless, these findings warrant further validation through large-scale prospective studies with extended follow-up periods to better establish causality and long-term implications.

## Supporting information

S1 Table Comparison of basic characteristics between excluded and included populations. (DOCX)

S2 Table Univariate analysis to assess the association of riboflavin intake with Helicobacter pylori seropositivity. (DOCX)

S3 Table Association between dietary riboflavin intake and Helicobacter pylori seropositivity in participants with extreme energy intake was not included. (DOCX)

S4 Table Population characteristics by categories of dietary riboflavin intake based on multiple imputation. (DOCX)

S5 Table Univariate analysis to assess the association of riboflavin intake with Helicobacter pylori seropositivity based on multiple imputation. (DOCX)

S6 Table Multivariable logistic regression to assess the association of riboflavin intake with Helicobacter pylori seropositivity based on multiple imputation. (DOCX)

S7 Table Raw data. (CSV)
