# Supplementary material for: The reverse association between riboflavin intake and Helicobacter pylori infection in US adults: A cross-sectional study
Source: PLoS One. 2025 Jun 30;20(6):e0326787. doi: 10.1371/journal.pone.0326787 (PMC12208485; doi:10.1371/journal.pone.0326787)
Supplement: S1 Table — (DOCX) [file pone.0326787.s001.docx]

Table S1. Comparison of basic characteristics between excluded and included populations.

| Characteristic | Total (n = 9965) | | Excluded population  (n = 7070) | Included population  (n = 2895) | p-Value |
| --- | --- | --- | --- | --- | --- |
| Age(year), Mean(SD) | 29.7 ± 24.8 | | 21.5 ± 22.2 | 49.9 ± 18.7 | < 0.001 |
| Sex, n (%) |  | |  |  | 0.049 |
| Male | 4883 (49.0) | | 3509 (49.6) | 1374 (47.5) |  |
| Female | 5082 (51.0) | | 3561 (50.4) | 1521 (52.5) |  |
| Education level (year),n(%) |  | |  |  | < 0.001 |
| <12 | 1918 (39.3) | | 875 (44.1) | 1043 (36) |  |
| =12 | 1097 (22.5) | | 438 (22.1) | 659 (22.8) |  |
| >12 | 1863 (38.2) | | 670 (33.8) | 1193 (41.2) |  |
| Family income, n (%) |  | |  |  | < 0.001 |
| Low | 3428 (40.4) | | 2560 (45.8) | 868 (30) |  |
| Medium | 2980 (35.1) | | 1887 (33.8) | 1093 (37.8) |  |
| High | 2074 (24.5) | | 1140 (20.4) | 934 (32.3) |  |
| Marital status, n (%) |  | |  |  | < 0.001 |
| Living alone | 3355 (55.3) | | 2287 (72) | 1068 (36.9) |  |
| Married or living with a partner | 2717 (44.7) | | 890 (28) | 1827 (63.1) |  |
| Body mass index(kg/m2),n(%) | | |  |  | < 0.001 |
| <25 | 4648 (54.9) | | 3751 (67.4) | 897 (31) |  |
| ≥25,<30 | 2039 (24.1) | | 998 (17.9) | 1041 (36) |  |
| ≥30 | 1775 (21.0) | | 818 (14.7) | 957 (33.1) |  |
| Smoker, n (%) | 2299 (47.1) | | 915 (46.2) | 1384 (47.8) | 0.259 |
| Drinker, n (%) | 2753 (66.2) | | 805 (63.6) | 1948 (67.3) | 0.02 |
| Diabetes, n (%) | 489 ( 5.2) | | 220 (3.3) | 269 (9.3) | < 0.001 |
| Hypertension, n (%) | 1532 (25.7) | | 641 (21) | 891 (30.8) | < 0.001 |
| Heart failure, n (%) | 167 ( 3.4) | | 82 (4.1) | 85 (2.9) | 0.024 |
| Coronary disease, n (%) | 203 ( 4.2) | | 89 (4.5) | 114 (3.9) | 0.348 |
| Angina, n (%) | 180 ( 3.7) | | 61 (3.1) | 119 (4.1) | 0.059 |
| Heart attack, n (%) | 221 ( 4.5) | | 96 (4.8) | 125 (4.3) | 0.392 |
| Stroke, n (%) | 190 ( 3.9) | | 101 (5.1) | 89 (3.1) | < 0.001 |
| Serum indicators |  | |  |  |  |
| Creatinine(mg/dL), Median (IQR) | | 0.6 (0.5, 0.8) | 0.6 (0.5, 0.7) | 0.7 (0.6, 0.9) | < 0.001 |
| C reactive protein (mg/dl), Median (IQR) | 0.1 (0.0, 0.4) | | 0.1 (0.0, 0.3) | 0.3 (0.1, 0.6) | < 0.001 |
| Albumin  (g/dL), Mean ± SD | 4.5 ± 0.4 | | 4.6 ± 0.4 | 4.4 ± 0.3 | < 0.001 |
| Total cholesterol (mg/dL),Mean±SD | 185.0 ± 43.0 | | 172.9 ± 42.2 | 198.9 ± 39.7 | < 0.001 |
| Riboflavin intake (mg/d),Mean ± SD | 1.9 ± 1.1 | | 1.9 ± 1.1 | 1.9 ± 1.1 | 0.915 |
| Calorie consumption (kcal/d), Mean ± SD | 1975.5 ± 990.1 | | 1927.5 ± 976.6 | 2072.3 ± 1010.0 | < 0.001 |
| Carbohydrate consumption (gm/d),Median (IQR) | 235.1 (165.3, 323.7) | | 233.3 (164.1, 322.5) | 237.7 (166.8, 325.4) | 0.114 |
| Dietary fiber consumption (gm/d), Median (IQR) | 11.5 (7.1, 17.6) | | 10.7 (6.6, 16.4) | 13.4 (8.7, 20.2) | < 0.001 |
| Dietary supplements taken  n (%) | 3595 (36.1) | | 2126 (30.1) | 1469 (50.7) | < 0.001 |
| VitaminB1 intake (mg/d), Median (IQR) | 1.3 (0.9, 1.9) | | 1.3 (0.9, 1.9) | 1.4 (0.9, 2.0) | < 0.001 |
| VitaminB6 intake (mg/d), Median (IQR) | 1.4 (0.9, 2.1) | | 1.4 (0.9, 2.1) | 1.6 (1.0, 2.3) | < 0.001 |
| VitaminC intake (mg/d), Median (IQR) | 74.1 (33.8, 140.5) | | 75.5 (34.8, 140.7) | 71.1 (31.7, 139.9) | 0.066 |
| VitaminA intake (RE/d), Median (IQR) | 582.9 (327.3,1020.9) | | 565.3 (323.3, 959.2) | 625.7 (333.1, 1153.6) | < 0.001 |
| Carotene intake (RE/d), Median (IQR) | 142.2 (61.3, 372.9) | | 122.3 (55.0, 306.0) | 191.7 (78.4, 514.4) | < 0.001 |
| VitaminE intake (mg/d), Median (IQR) | 6.5 (4.1, 9.8) | | 6.2 (4.0, 9.4) | 7.0 (4.5, 10.6) | < 0.001 |
| Niacin intake (mg/d), Median (IQR) | 17.6 (11.9, 25.6) | | 16.8 (11.2, 24.5) | 19.5 (13.5, 27.7) | < 0.001 |
| Folate intake (mcg/d), Median (IQR) | 296.9 (191.9, 435.5) | | 284.0 (182.6, 420.3) | 320.8 (213.9, 467.0) | < 0.001 |
| VitaminB12 intake (mcg/d), Median (IQR) | 3.1 (1.8, 5.0) | | 3.0 (1.7, 4.9) | 3.3 (1.8, 5.3) | < 0.001 |
| Calcium intake (mg/d), Median (IQR) | 697.1 (437.7, 1067.7) | | 717.6 (453.6, 1079.9) | 654.9 (403.9, 1037.1) | < 0.001 |
| Phosphorus intake (mg/d), Median (IQR) | 1068.1(740.1,1489.5) | | 1035.8 (714.0, 1437.9) | 1127.3 (798.7, 1574.9) | < 0.001 |
| Iron intake (mg/d), Median (IQR) | 12.5 (8.6, 18.2) | | 12.4 (8.4, 18.1) | 12.7 (8.8, 18.4) | 0.024 |
| Zinc intake (mg/d), Median (IQR) | 8.9 (6.0, 13.2) | | 8.7 (5.8, 12.9) | 9.3 (6.3, 13.5) | < 0.001 |
| Sodium intake (mg/d), Median (IQR) | 2721.2 (1829.3, 3904.4) | | 2634.2 (1743.6, 3798.2) | 2916.1 (1987.2, 4141.7) | < 0.001 |
| Potassium intake (mg/d), Median (IQR) | 2191.4 (1491.0, 3085.1) | | 2059.2 (1399.5, 2909.6) | 2487.2 (1724.9, 3409.2) | < 0.001 |
| H.pylori seropositivity n (%) | 2674 (35.7) | | 1394 (30.3) | 1280 (44.2) | < 0.001 |
