# Supplementary material for: The reverse association between riboflavin intake and Helicobacter pylori infection in US adults: A cross-sectional study
Source: PLoS One. 2025 Jun 30;20(6):e0326787. doi: 10.1371/journal.pone.0326787 (PMC12208485; doi:10.1371/journal.pone.0326787)
Supplement: S2 Table — (DOCX) [file pone.0326787.s002.docx]

**Table S2.** Univariate analysis to assess the association of riboflavin intake with Helicobacter pylori seropositivity.

| Variable | OR(95%CI) | | p-value |  | Variable | OR(95%CI) | | | p-value |
| --- | --- | --- | --- | --- | --- | --- | --- | --- | --- |
| Age(year) | 1.02 (1.01~1.02) | | <0.001 |  | **Serum indicators** |  | | |  |
| Sex, n (%) |  | |  |  | Creatinine (mg/dL) | 0.97 (0.86~1.1) | | | 0.677 |
| Male | 1(reference) | |  |  | C reactive protein (mg/dL) | 1.04(0.96~1.12) | | | 0.373 |
| Female | 0.91(0.78~1.05) | | 0.19 |  | Albumin (g/dL) | 0.73 (0.59~0.9) | | | 0.004 |
| Education level (year),n(%) |  | |  |  | Total cholesterol (mg/dL) | 1 (1~1) | | | 0.25 |
| <12 | 1(reference) | |  |  |  |  | | |  |
| =12 | 0.3 (0.24~0.36) | | <0.001 |  | **Dietary information** | | |  |  |
| >12 | 0.18 (0.15~0.22) | | <0.001 |  | Calorie consumption (kcal/d) | 1 (1~1) | | | <0.001 |
| Marital status, n (%) |  | |  |  | Carbohydrate consumption(gm/d) | | 1 (1~1) | | <0.001 |
| Living alone | 1(reference) | |  |  | Dietary fiber consumption(gm/d) | 0.99 (0.99~1) | | | 0.082 |
| Married or living with a partner | | 1.09(0.94~1.27) | 0.271 |  | Dietary supplements taken, n(%) | 0.54(0.46~0.62) | | | <0.001 |
| Family income, n (%) |  | |  |  | VitaminB1 intake (mg/d) | 0.82 (0.76~0.9) | | | <0.001 |
| Low | 1(reference) | |  |  | VitaminB6 intake (mg/d) | 0.87(0.81~0.93) | | | <0.001 |
| Medium | 0.59 (0.5~0.71) | | <0.001 |  | Vitamin C intake (mg/d) | 1 (1~1) | | | 0.989 |
| High | 0.26 (0.21~0.32) | | <0.001 |  | Vitamin A intake (RE/d) | 1 (1~1) | | | 0.001 |
| Body mass index(kg/m2),n(%) |  | |  |  | Carotene intake (RE/d) | 1 (1~1) | | | 0.259 |
| <25 | 1(reference) | |  |  | Vitamin E intake (mg/d) | 0.97(0.96~0.98) | | | <0.001 |
| ≥25,<30 | 1.3 (1.09~1.56) | | 0.004 |  | Niacin intake (mg/d) | 0.98(0.98~0.99) | | | <0.001 |
| ≥30 | 1.26 (1.05~1.51) | | 0.014 |  | Folate intake (mcg/d) | 1 (1~1) | | | 0.004 |
| Smoker, n(%) | 1.17 (1.01~1.36) | | 0.035 |  | VitaminB12 intake (mcg/d) | 0.98 (0.97~1) | | | 0.008 |
| Drinker, n(%) | 0.81 (0.69~0.95) | | 0.008 |  | Calcium intake (mg/d) | 1 (1~1) | | | <0.001 |
| Diabetes, n(%) | 2.08 (1.61~2.68) | | <0.001 |  | Phosphorus intake (mg/d) | 1 (1~1) | | | <0.001 |
| Hypertension, n(%) | 1.31 (1.12~1.53) | | 0.001 |  | Iron intake (mg/d) | 0.98(0.97~0.99) | | | <0.001 |
| Heart failure, n(%) | 1.75 (1.13~2.7) | | 0.012 |  | Zinc intake (mg/d) | 0.98(0.97~0.99) | | | <0.001 |
| Coronary disease, n(%) | 1.14 (0.78~1.66) | | 0.489 |  | Sodium intake (mg/d) | 1 (1~1) | | | <0.001 |
| Angina, n(%) | 1.34 (0.93~1.94) | | 0.115 |  | Potassium intake (mg/d) | 1 (1~1) | | | <0.001 |
| Heart attack, n(%) | 1.43 (1~2.05) | | 0.049 |  | Riboflavin intake (mg/d) | 0.78(0.73~0.84) | | | <0.001 |
| Stroke, n(%) | 1.72 (1.12~2.64) | | 0.013 |  |  |  | | |  |
