# Supplementary material for: The reverse association between riboflavin intake and Helicobacter pylori infection in US adults: A cross-sectional study
Source: PLoS One. 2025 Jun 30;20(6):e0326787. doi: 10.1371/journal.pone.0326787 (PMC12208485; doi:10.1371/journal.pone.0326787)
Supplement: S3 Table — (DOCX) [file pone.0326787.s003.docx]

**TableS3.** Association between dietary riboflavin intake and H.pylori seropositivity in participants with extreme energy intake was not included

| Variable |  |  |  | OR(95%CI ) |  |  |  |  |
| --- | --- | --- | --- | --- | --- | --- | --- | --- |
|  | Crude | p-value | Model1 | p-value | Model 2 | p-value | Model3 | p-value |
| Riboflavin intake(mg/day) | | |  |  |  |  |  |  |
|  | 0.77 (0.71~0.83) | <0.001 | 0.84 (0.77~0.91) | <0.001 | 0.83 (0.76~0.9) | <0.001 | 0.94 (0.75~1.17) | 0.57 |
| Q1(≤1.13) | 1(Ref) |  | 1(Ref) |  | 1(Ref) |  | 1(Ref) |  |
| Q2(1.14- 1.64) | 0.73 (0.59~0.9) | 0.003 | 0.8 (0.64~1.01) | 0.059 | 0.79 (0.63~1) | 0.047 | 0.83 (0.65~1.07) | 0.155 |
| Q3(1.65- 2.34) | 0.57 (0.46~0.7) | <0.001 | 0.67 (0.53~0.85) | 0.001 | 0.66 (0.52~0.83) | 0.001 | 0.72 (0.53~0.97) | 0.033 |
| Q4(≥2.35) | 0.46 (0.37~0.57) | <0.001 | 0.58 (0.46~0.74) | <0.001 | 0.57 (0.45~0.73) | <0.001 | 0.74 (0.47~1.15) | 0.177 |
| Trend test | 0.77 (0.72~0.83) | <0.001 |  | <0.001 |  | <0.001 |  | 0.068 |

Q, quartiles; OR, odds ratio; CI, confidence interval; Ref: reference.

Model 1: Adjusted for sociodemographic variables (age, sex, education level, marital status, family income);

Model 2: Model 1 + lifestyle and clinical variables (BMI, smoking status, alcohol consumption, diabetes, cardiovascular diseases) and serum biomarkers (creatinine, CRP, albumin, total cholesterol)

Model 3: Model 2 + dietary covariates (total caloric intake, carbohydrates, dietary fiber, vitamins B1/B6/B12/C/A/E, carotene, niacin, folate, calcium, phosphorus, iron, zinc, sodium, potassium, and dietary supplements).
