# Supplementary material for: The reverse association between riboflavin intake and Helicobacter pylori infection in US adults: A cross-sectional study
Source: PLoS One. 2025 Jun 30;20(6):e0326787. doi: 10.1371/journal.pone.0326787 (PMC12208485; doi:10.1371/journal.pone.0326787)
Supplement: S4 Table — (DOCX) [file pone.0326787.s004.docx]

**TableS4.** Population characteristics by categories of dietary riboflavin intake based on multiple imputation.

| Characteristic | Riboflavin intake, mg/d | | | | | |
| --- | --- | --- | --- | --- | --- | --- |
|  | Total | Q1(≤1.13) | Q2(1.14-1.64) | Q3(1.65-2.34) | Q4（≥2.35) | p-Value |
| NO. | 4000 | 984 | 1013 | 998 | 1005 |  |
| Age (year), Mean (SD) | 49.8 ± 18.7 | 51.4 ± 18.6 | 51.1 ± 18.6 | 50.0 ± 18.7 | 46.8 ± 18.7 | < 0.001 |
| Sex, n (%) |  |  |  |  |  | < 0.001 |
| Male | 1871 (46.8) | 332 (33.7) | 409 (40.4) | 513 (51.4) | 617 (61.4) |  |
| Female | 2129 (53.2) | 652 (66.3) | 604 (59.6) | 485 (48.6) | 388 (38.6) |  |
| Education level (year), n (%) | |  |  |  |  | < 0.001 |
| <12 | 1543 (38.6) | 502 (51) | 414 (40.9) | 344 (34.5) | 283 (28.2) |  |
| =12 | 897 (22.4) | 200 (20.3) | 237 (23.4) | 231 (23.1) | 229 (22.8) |  |
| >12 | 1560 (39.0) | 282 (28.7) | 362 (35.7) | 423 (42.4) | 493 (49.1) |  |
| Family income, n (%) | |  |  |  |  | < 0.001 |
| Low | 1230 (30.8) | 383 (38.9) | 325 (32.1) | 271 (27.2) | 251 (25) |  |
| Medium | 1527 (38.2) | 383 (38.9) | 394 (38.9) | 383 (38.4) | 367 (36.5) |  |
| High | 1243 (31.1) | 218 (22.2) | 294 (29) | 344 (34.5) | 387 (38.5) |  |
| Marital status, n (%) | |  |  |  |  | < 0.001 |
| Living alone | 1500 (37.5) | 452 (45.9) | 366 (36.1) | 359 (36) | 323 (32.1) |  |
| Married or living with a partner | 2500 (62.5) | 532 (54.1) | 647 (63.9) | 639 (64) | 682 (67.9) |  |
| Body mass index (kg/m2), n(%) | |  |  |  |  | 0.008 |
| <25 | 1272 (31.8) | 291 (29.6) | 311 (30.7) | 313 (31.4) | 357 (35.5) |  |
| ≥25,<30 | 1417 (35.4) | 342 (34.8) | 376 (37.1) | 338 (33.9) | 361 (35.9) |  |
| ≥30 | 1311 (32.8) | 351 (35.7) | 326 (32.2) | 347 (34.8) | 287 (28.6) |  |
| Smoker, n (%) | 1875 (46.9) | 405 (41.2) | 462 (45.6) | 526 (52.7) | 482 (48) | < 0.001 |
| Drinker, n (%) | 2640 (66.0) | 554 (56.3) | 655 (64.7) | 709 (71) | 722 (71.8) | < 0.001 |
| Diabetes, n (%) | 381 (9.5) | 100 (10.2) | 108 (10.7) | 101 (10.1) | 72 (7.2) | 0.031 |
| Hypertension, n (%) | 1212 (30.3) | 335 (34) | 341 (33.7) | 306 (30.7) | 230 (22.9) | < 0.001 |
| Heart failure, n (%) | 121 (3.0) | 46 (4.7) | 30 (3) | 25 (2.5) | 20 (2) | 0.003 |
| Coronary disease, n (%) | 158 (4.0) | 42 (4.3) | 43 (4.2) | 40 (4) | 33 (3.3) | 0.641 |
| Angina, n (%) | 147 (3.7) | 34 (3.5) | 44 (4.3) | 38 (3.8) | 31 (3.1) | 0.484 |
| Heart attack, n (%) | 174 (4.3) | 46 (4.7) | 51 (5) | 41 (4.1) | 36 (3.6) | 0.399 |
| Stroke, n (%) | 129 (3.2) | 42 (4.3) | 29 (2.9) | 33 (3.3) | 25 (2.5) | 0.131 |
| Serum indicators |  |  |  |  |  |  |
| C reactive protein  (mg/dl), Median (IQR) | 0.3 (0.1, 0.6) | 0.3 (0.1, 0.7) | 0.3 (0.1, 0.6) | 0.2 (0.1, 0.5) | 0.2 (0.1, 0.5) | < 0.001 |
| Creatinine  (mg/dL), Median (IQR) | 0.7 (0.6, 0.8) | 0.7 (0.5, 0.8) | 0.7 (0.5, 0.8) | 0.7 (0.6, 0.9) | 0.7 (0.6, 0.9) | < 0.001 |
| Albumin  (g/dL), Mean ± SD | 4.4 ± 0.4 | 4.4 ± 0.3 | 4.4 ± 0.3 | 4.4 ± 0.3 | 4.4 ± 0.4 | < 0.001 |
| Total cholesterol  (mg/dL), Mean ± SD | 199.6 ± 40.8 | 200.5 ± 42.4 | 199.8 ± 40.1 | 199.2 ± 40.1 | 198.7 ± 40.7 | 0.776 |
| Dietary supplements taken, n (%) | 2005 (50.1) | 405 (41.2) | 501 (49.5) | 513 (51.4) | 586 (58.3) | < 0.001 |
| Calorie consumption  (kcal/d), Mean ± SD | 1872.3(1383.2, 2556.4) | 1177.9(898.3, 1538.8) | 1694.4(1377.0, 2103.4) | 2141.3(1694.0, 2622.6) | 2796.2(2246.4, 3563.7) | < 0.001 |
| Carbohydrate consumption  (gm/d), Median (IQR) | 236.0 (166.3, 325.9) | 158.0(110.3, 206.8) | 211.1(159.3, 270.6) | 261.4(200.5, 328.2) | 351.2(278.4, 449.6) | < 0.001 |
| Dietary fiber consumption  (gm/d), Median (IQR) | 13.5 (8.5, 20.1) | 8.3 (5.2, 12.6) | 12.6 (8.5, 18.0) | 14.8 (10.1, 20.7) | 20.2(14.1,28.7) | < 0.001 |
| VitaminB1 intake  (mg/d), Median (IQR) | 1.4 (0.9, 2.0) | 0.8 (0.6, 1.0) | 1.2 (0.9, 1.5) | 1.6 (1.3, 1.9) | 2.4 (1.9, 3.0) | < 0.001 |
| VitaminB6 intake  (mg/d), Median (IQR) | 1.6 (1.0, 2.3) | 0.9 (0.6, 1.3) | 1.4 (1.0, 1.8) | 1.8 (1.3, 2.3) | 2.7 (2.1, 3.6) | < 0.001 |
| VitaminC intake  (mg/d), Median (IQR) | 71.9(31.6, 142.1) | 40.6 (15.0, 93.0) | 65.7(29.6, 128.7) | 75.7(37.1, 148.5) | 111.7(59.3, 202.2) | < 0.001 |
| VitaminA intake]  (RE/d), Median (IQR) | 617.9 (328.0, 1140.7) | 260.8 (143.9, 507.9) | 468.0 (321.3, 774.6) | 698.1 (486.1, 1112.4) | 1196.7 (786.3, 1939.2) | < 0.001 |
| Carotene intake  (RE/d), Median (IQR) | 187.4 (76.8, 517.5) | 104.0 (38.9, 319.0) | 177.2 (72.0, 464.9) | 216.2 (92.3, 609.7) | 264.1 (129.4, 724.3) | < 0.001 |
| VitaminE intake  (mg/d), Median (IQR) | 6.9 (4.4, 10.5) | 4.2 (2.6, 6.0) | 6.2 (4.4, 8.9) | 7.9 (5.6, 11.0) | 10.7 (7.2, 16.1) | < 0.001 |
| Niacin intake  (mg/d), Median (IQR) | 19.3(13.3, 27.4) | 11.9 (8.2, 15.6) | 16.6 (13.0, 21.9) | 21.4 (16.5, 27.2) | 31.1(23.7,40.7) | < 0.001 |
| Folate intake  (mcg/d), Median (IQR) | 319.6 (210.9, 465.7) | 178.3 (126.2, 246.4) | 272.3 (208.1, 360.2) | 359.6 (279.9, 453.2) | 536.7 (411.2, 703.5) | < 0.001 |
| VitaminB12 intake  (mcg/d), Median (IQR) | 3.3 (1.8, 5.3) | 1.5 (0.8, 2.7) | 2.6 (1.7, 3.9) | 3.8 (2.5, 5.4) | 6.0 (4.1, 9.2) | < 0.001 |
| Calcium intake  (mg/d), Median (IQR) | 651.1 (405.0, 1030.5) | 308.6 (197.3, 432.9) | 547.7 (416.2, 703.0) | 805.6 (615.5, 1031.9) | 1264.1 (952.7, 1630.5) | < 0.001 |
| Phosphorus intake  (mg/d), Median (IQR) | 1127.3 (798.0, 1567.0) | 628.5 (476.0, 775.5) | 982.7 (830.7, 1147.3) | 1293.4 (1097.4, 1530.2) | 1857.1(1550.1, 2248.3) | < 0.001 |
| Iron intake  (mg/d), Median (IQR) | 12.6 (8.8, 18.6) | 7.4 (5.4, 9.5) | 11.0 (8.8, 13.9) | 14.4 (11.3, 18.6) | 22.4(16.9,29.8) | < 0.001 |
| Zinc intake  (mg/d), Median (IQR) | 9.3 (6.3, 13.6) | 5.1 (3.6, 7.2) | 8.1 (6.3, 10.8) | 10.5 (8.2, 13.8) | 15.6(11.9,21.3) | < 0.001 |
| Sodium intake  (mg/d), Median (IQR) | 2901.7 (1980.2, 4145.5) | 1783.4 (1194.1, 2440.1) | 2604.4 (1935.3, 3414.2) | 3333.2 (2502.1, 4271.0) | 4401.1(3249.6, 5854.7) | < 0.001 |
| Potassium intake  (mg/d), Median (IQR) | 2471.9 (1726.1, 3407.2) | 1458.4 (1032.7, 1992.8) | 2205.9 (1720.9, 2787.6) | 2788.9 (2215.0, 3385.9) | 3807.1(3041.4, 4711.5) | < 0.001 |
| H.pylori seropositivity, n (%) | 1842 (46.1) | 555 (56.4) | 499 (49.3) | 414 (41.5) | 374 (37.2) | < 0.001 |
