# Supplementary material for: The reverse association between riboflavin intake and Helicobacter pylori infection in US adults: A cross-sectional study
Source: PLoS One. 2025 Jun 30;20(6):e0326787. doi: 10.1371/journal.pone.0326787 (PMC12208485; doi:10.1371/journal.pone.0326787)
Supplement: S5 Table — (DOCX) [file pone.0326787.s005.docx]

**Table S5.** Univariate analysis to assess the association of riboflavin intake with Helicobacter pylori seropositivity based on multiple imputation.

| Variable | OR(95%CI) | P value |  | Variable | | OR(95%CI) | P value |
| --- | --- | --- | --- | --- | --- | --- | --- |
| Age(year) | 1.02 (1.01~1.02) | <0.001 |  | **Serum indicators** | |  |  |
| Sex, n (%) |  |  |  | C reactive protein (mg/dL) | | 1.07 (1~1.14) | 0.051 |
| Male | 1(reference) | |  | Albumin (g/dL) | | 0.72 (0.6~0.86) | <0.001 |
| Female | 0.88 (0.78~1) | 0.046 |  | Creatinine (mg/dL) | | 0.99 (0.89~1.1) | 0.889 |
| Education level (year),n(%) | | |  | Total cholesterol (mg/dL) | | 1 (1~1) | 0.132 |
| <12 | 1(reference) | |  |  | |  |  |
| =12 | 0.31 (0.26~0.37) | <0.001 |  | **Dietary information** | |  |  |
| >12 | 0.19 (0.17~0.23) | <0.001 |  | Dietary supplements taken,  n(%) | | 0.56 (0.5~0.64) | <0.001 |
| Family income, n (%) | |  |  | Calorie consumption (kcal/d) | | 1 (1~1) | <0.001 |
| Low | 1(reference) | |  | Carbohydrate consumption  (gm/d) | | 1 (1~1) | <0.001 |
| Medium | 0.61 (0.52~0.71) | <0.001 |  | Dietary fiber consumption  (gm/d) | | 0.99 (0.99~1) | 0.03 |
| High | 0.28 (0.24~0.33) | <0.001 |  | VitaminB1 intake (mg/d) | | 0.8 (0.75~0.86) | <0.001 |
| Marital status, n (%) | |  |  | VitaminB6 intake (mg/d) | | 0.86 (0.81~0.91) | <0.001 |
| Living alone | 1(reference) | |  | Vitamin C intake (mg/d) | | 1 (1~1) | 0.862 |
| Married or living with a partner | 1.11 (0.97~1.26) | 0.12 |  | Vitamin A intake (RE/d) | | 1 (1~1) | <0.001 |
| Body mass index(kg/m2),n(%) | | |  | Carotene intake (RE/d) | | 1 (1~1) | 0.158 |
| <25 | 1(reference) | |  | Vitamin E intake (mg/d) | | 0.97 (0.96~0.98) | <0.001 |
| ≥25,<30 | 1.29 (1.11~1.5) | 0.001 |  | Niacin intake (mg/d) | | 0.98 (0.98~0.99) | <0.001 |
| ≥30 | 1.21(1.04~1.41) | 0.017 |  | Folate intake (mcg/d) | | 1 (1~1) | <0.001 |
| Smoker, n (%) | 1.13 (1~1.28) | 0.052 |  | VitaminB12 intake (mcg/d) | | 0.99 (0.98~1) | 0.022 |
| Drinker, n(%) | 0.79 (0.69~0.9) | <0.001 |  | Calcium intake (mg/d) | | 1 (1~1) | <0.001 |
| Diabetes, n(%) | 1.87 (1.51~2.32) | <0.001 |  | Phosphorus intake (mg/d) | | 1 (1~1) | <0.001 |
| Hypertension, n(%) | 1.35 (1.18~1.54) | <0.001 |  | Iron intake (mg/d) | 0.98(0.97~0.98) | | <0.001 |
| Heart failure, n(%) | 1.95 (1.34~2.83) | <0.001 |  | Zinc intake (mg/d) | | 0.99 (0.98~1) | 0.014 |
| Coronary disease, n(%) | 1.31 (0.95~1.8) | 0.096 |  | Sodium intake (mg/d) | | 1 (1~1) | <0.001 |
| Angina, n(%) | 1.34 (0.96~1.86) | 0.083 |  | Potassium intake (mg/d) | | 1 (1~1) | <0.001 |
| Heart attack, n(%) | 1.47 (1.08~1.99) | 0.014 |  | Riboflavin intake (mg/d) | 0.77(0.72~0.82) | | <0.001 |
| Stroke, n(%) | 1.45 (1.02~2.07) | 0.038 |  |  | |  |  |
