# Supplementary material for: The reverse association between riboflavin intake and Helicobacter pylori infection in US adults: A cross-sectional study
Source: PLoS One. 2025 Jun 30;20(6):e0326787. doi: 10.1371/journal.pone.0326787 (PMC12208485; doi:10.1371/journal.pone.0326787)
Supplement: S6 Table — (DOCX) [file pone.0326787.s006.docx]

**Table S6.**Multivariable logistic regression to assess the association of riboflavin intake with Helicobacter pylori seropositivity based on multiple imputation.

| Variable |  |  |  | OR(95%CI ) | |  |  |  |  |
| --- | --- | --- | --- | --- | --- | --- | --- | --- | --- |
|  | Crude | p-value | Model1 | | p-value | Model 2 | p-value | Model3 | p-value |
| Riboflavin intake(mg/day) | | |  | |  |  |  |  |  |
|  | 0.77  (0.72~0.82) | <0.001 | 0.84  (0.79~0.9) | | <0.001 | 0.83  (0.78~0.89) | <0.001 | 0.9  (0.75~1.09) | 0.283 |
| Q1(≤1.13) | 1(Ref) |  | 1(Ref) | |  | 1(Ref) |  | 1(Ref) |  |
| Q2(1.14- 1.64) | 0.75  (0.63~0.9) | 0.001 | 0.83  (0.69~1) | | 0.054 | 0.82  (0.68~1) | 0.048 | 0.87  (0.71~1.07) | 0.189 |
| Q3(1.65- 2.34) | 0.55  (0.46~0.65) | <0.001 | 0.65  (0.53~0.79) | | <0.001 | 0.64  (0.53~0.78) | <0.001 | 0.73  (0.57~0.92) | 0.009 |
| Q4(≥2.35) | 0.46  (0.38~0.55) | <0.001 | 0.6  (0.49~0.73) | | <0.001 | 0.59  (0.48~0.72) | <0.001 | 0.78  (0.55~1.11) | 0.166 |
| Trend test | 0.77  (0.72~0.81) | <0.001 | 0.84  (0.79~0.89) | | <0.001 | 0.83  (0.78~0.89) | <0.001 | 0.89  (0.8~0.99) | 0.037 |

Q, quartiles; OR, odds ratio; CI, confidence interval; Ref: reference.

Model 1: Adjusted for sociodemographic variables (age, sex, education level, marital status, family income);

Model 2: Model 1 + lifestyle and clinical variables (BMI, smoking status, alcohol consumption, diabetes, cardiovascular diseases) and serum biomarkers (creatinine, CRP, albumin, total cholesterol)

Model 3: Model 2 + dietary covariates (total caloric intake, carbohydrates, dietary fiber, vitamins B1/B6/B12/C/A/E, carotene, niacin, folate, calcium, phosphorus, iron, zinc, sodium, potassium, and dietary supplements).
